# HbA1c or fructosamine on evaluating glucose intolerance in children with beta- thalassemia

**DOI:** 10.1038/s41390-024-03146-y

**Published:** 2024-04-04

**Authors:** Asmaa A. Mahmoud, Mahmoud A. El-Hawy, Esraa T. Allam, Ahmed H. Salem, Ahmed S. Abo Hola

**Affiliations:** 1 Department of Pediatrics, Faculty of Medicine, Menoufia University, Shebin El-Kom, Egypt; 2 Department of Clinical Pathology, National Liver Institute, Shebin El-Kom, Egypt; 3 Egyptian Ministry of Health, Cairo, Egypt

## Abstract

**Background:**

Beta-thalassemia major (β-TM) patients are more likely to experience blood glucose intolerance and to date; the blood markers that could evaluate this are debatable. So, this study aimed to assess the roles of glycated hemoglobin A1c (HbA1c) and fructosamine in evaluating glucose intolerance in children with β-TM and figuring out role of insulin resistance in these patients.

**Methods:**

One hundred children diagnosed with β-TM and 100 age and sex-matched controls were enrolled. Fasting plasma glucose (FPG), 2-h post-prandial blood glucose (2-h PG), HbA1c, fructosamine, fasting insulin level (FINS), insulin resistance index (HOMA-IR), and insulin sensitivity index (HOMA-IS) were evaluated.

**Results:**

FPG and 2-h PG revealed glucose intolerance in 51 patients (51%), 19 of them had diabetes mellitus. HbA1c, fructosamine, FINS, and HOMA-IR showed a high statistically significant increase in patients compared to controls, (*P* < 0.001). Results revealed fructosamine was more specific in detecting prediabetes state and more sensitive in identifying diabetes mellitus in our patients when compared to HbA1c.

**Conclusions:**

Despite controversies on HbA1c in children with β-TM, it is still valuable in glucose intolerance detection. Fructosamine showed more sensitivity and specificity. Furthermore, insulin resistance was prevalent in children with β-TM highlighting the necessity of regular glycemic state evaluation.

**Impact:**

- Glucose intolerance is a common complication in beta thalassemia patients.
- Conflicting data was reported about the role of HbA1c and fructosamine in evaluating glucose intolerance in thalassemic patients.
- Fructosamine does not yet have a threshold that may be used to distinguish between patients who have diabetes mellitus and those who do not.
- Fructosamine was more specific in detecting blood glucose intolerance compared to HbA1c and was more sensitive for diagnosing diabetes mellitus.
- Insulin resistance was common in patients with beta-thalassemia and often present before the onset of overt diabetes.

## Introduction

Beta-thalassemia major (β-TM) is the most common genetic disorder of hemoglobinopathies characterized by reduced or absent production of the beta-globin chain that results in ineffective erythropoiesis which results in chronic hemolytic anemia and blood transfusion dependency.^1^

Iron overload related to regular blood transfusion can cause multiple organ damage like cardiomyopathy, liver cirrhosis, arthritis, and endocrine disorders such as diabetes mellitus. Moreover, older age, more frequent blood transfusions, the type and dosage of iron-chelating therapy, high serum ferritin, a family history of diabetes mellitus, hepatic impairment, and genetic iron overload modifiers are suggested risk factors for the development of diabetes mellitus in individuals with β-TM.^2^

A meta-analysis study reported a prevalence of 17.21%, 12.46%, and 6.54% for impaired fasting glucose, impaired glucose tolerance and established diabetes mellitus in patients with β-TM, respectively.^3^ However, the precise mechanisms causing the transition from normoglycemia to overt diabetes are still not well known. A growing amount of research shows that both insulin resistance and the direct toxic effects of iron on pancreatic beta cells and their insulin secretory ability are both involved in the development of diabetes mellitus with β-TM.^4^

It’s well known that hemoglobin A1c (HbA1c) can reflect the average blood glucose status from the previous 2 to 3 months, however, fructosamine a byproduct of the non-enzymatic interaction between glucose and proteins has been suggested as a substitute for HbA1c and can reflect blood glucose status over last 2 to 3 weeks. Additionally, the American Diabetes Association (ADA) suggests the use of fructosamine in patients with hemolytic anemia and hemoglobinopathies being known to be unaffected by erythrocyte life duration, erythropoiesis, and hemoglobin level.^5,6^

Another biomarker for short-term (2–3 weeks) glycemic control is glycated albumin (GA), a non-enzymatic glycation product of circulating albumin. GA is more reliable than Hb A1c to evaluate glycemic variability as its levels are not affected by any hemolytic processes, and more advantageous to fructosamine, as it is not influenced by other serum proteins.^7^ Also, GA may help the early detection of gestational diabetes mellitus with short-term glycemic monitoring and stratifying the risk of complications in these patients.^8,9^

Diverse studies offer conflicting perspectives on this matter, so this study aimed to evaluate glucose intolerance in children with β-TM and investigate the role of HbA1c, fructosamine, insulin resistance index (HOMA-IR), and insulin sensitivity index (HOMA-IS) in these patients.

## Methods

### Study design

A total of 100 children were diagnosed with β-TM by hemoglobin electrophoresis; in addition 100 age and sex-matched healthy children were enrolled in this study during the period from June 2022 to March 2023. Patients with a family history of diabetes mellitus, patients who had taken medications affecting glucose metabolism, obese patients, patients with other endocrine abnormalities, patients with hypoalbuminemia, associated non-thalassemia-related chronic illness or hereditary diseases and those who received marrow transplantation were excluded from the study. This study was approved by the ethical committee of the Faculty of Medicine, Menoufia University (ID 3/2022 PEDI 4).

All patients were subjected to detailed medical history including age, sex, consanguinity, disease duration, and blood transfusion frequency with transfusion index calculation (transfused packed red cell volume, expressed as the mean value for the previous two years, in milliliters per kilogram of body weight annually) and compliance to chelation therapy. The mean serum ferritin level of the previous six months was documented. Anthropometric measurements were recorded including weight, height, and body mass index (BMI) and a thorough clinical examination was done to assess the presence of any complications.

### Sample collection assay

Blood samples were withdrawn under aseptic conditions at 9:00 a.m. on an empty stomach to measure complete blood count (CBC) using Sysmex XT-1800i Automated Hematology Analyzer, serum ferritin, HbA1c and fructosamine and fasting plasma glucose (FPG) by using cobas c 311 auto analyzer, Roche diagnostics, Germany, and fasting insulin level (FINS) by using electrochemiluminescence immunoassay using cobas e 411 auto analyzer, Roche diagnostics, Germany. To measure 2-h post-prandial blood glucose, each patient received oral glucose at a dosage of 1.75 g/kg, up to a maximum of 75 g dissolved in 200 ml of water, and 2-h later, second blood glucose measurement was obtained.

In addition, the Homeostatic Model Assessment for insulin resistance (HOMA-IR) and for insulin sensitivity (HOMA-IS) was calculated.^6,10^

$${{{{{\rm{Insulin}}}}}}\,{{{{{\rm{resistance}}}}}}\,{{{{{\rm{index}}}}}}\,({{{{{\rm{HOMA}}}}}}-{{{{{\rm{IR}}}}}}) = 	 \, \{{{{{{\rm{fasting}}}}}}\,{{{{{\rm{insulin}}}}}}\,{{{{{\rm{level}}}}}}\,({{{{{\rm{mU}}}}}}/{{{{{\rm{L}}}}}})\\ 	 \times {{{{{\rm{fasting}}}}}}\,{{{{{\rm{plasma}}}}}}\,{{{{{\rm{glucose}}}}}}\,({{{{{\rm{mmol}}}}}}/{{{{{\rm{L}}}}}})\}/22.5$$

$${{{{{\rm{Insulin}}}}}}\,{{{{{\rm{sensitivity}}}}}}\,{{{{{\rm{index}}}}}}\,({{{{{\rm{HOMA}}}}}}-{{{{{\rm{IS}}}}}})=	 \, 1/ \big\{{{{{{\rm{fasting}}}}}}\,{{{{{\rm{insulin}}}}}}\,{{{{{\rm{level}}}}}} \,({{{{{\rm{mU}}}}}}/{{{{{\rm{L}}}}}})\\ 	 \times {{{{{\rm{fasting}}}}}}\,{{{{{\rm{plasma}}}}}}\,{{{{{\rm{glucose}}}}}}\,({{{{{\rm{mmol}}}}}}/{{{{{\rm{L}}}}}})$$

### Patients’ subgroups

The patients were classified into two major groups, glucose tolerant and glucose intolerant. The glucose intolerant group included prediabetes either with impaired fasting glucose level (fasting plasma glucose 100 mg/dl to 125 mg/dl) or with impaired glucose tolerance (2-h post-prandial blood glucose 140 mg/dl to 199 mg/dl), and diabetes mellitus (fasting plasma glucose ≥126 mg/dl or 2-h post-prandial blood glucose ≥200 mg/dl).^11^

### Sample size determination

Based on a review of past literature,^6^ the sample size was determined to be 72 subjects by using statistics and the Sample Size Pro tool version 6. As the power of the study was 80% and the confidence level was 95%, the sample size was increased to 100 subjects.

### Statistical analysis

Normally distributed quantitative data was presented as mean ± standard deviation (SD), while non-normally distributed one was presented as median (interquartile range ‘IQR’). In contrast to The Mann–Whitney *U* test, the *t*-test was used to compare the means of normally distributed quantitative data. The Chi-square test was used to assess the relationship between qualitative variables, and the Spearman correlation was used to examine correlations between the investigated data. The association between the three groups was analyzed using a one-way ANOVA with Bonferroni corrections. By using logistic regression, the correlation between numerous independent and dependent variables was examined. For all the data analyzed, a *p* < 0.05 was considered statistically significant. All the analysis was performed using IBM SPSS Statistics for Windows, Version 20.0. (IBM Corp, Armonk, NY).

## Results

Demographic, clinical criteria and laboratory data of the study groups were presented in Table 1. There was no statistically significant difference between patients and controls regarding age (11.49 ± 1.71 years versus 11.77 ± 2.30 years) and sex (males 59%, females 41% versus 55% and 45%) respectively. Positive consanguinity was highly statistically significant in patients compared to controls, (*p* < 0.001). Regarding the laboratory data, patients showed a highly statistically significant decrease in hemoglobin levels. A highly statistically significant increase in HbA1c, fructosamine, fasting plasma glucose (FPG), fasting insulin level (FINS), and insulin resistance index (HOMA-IR), was detected in patients compared to controls (*p* < 0.001). Conversely, insulin sensitivity index (HOMA-IS) was statistically lower in patients compared to controls (*p* < 0.001).

**Table 1:** Demographic, clinical criteria, and laboratory data of the studied groups.

Data	Patients (*n* = 100)	Controls (*n* = 100)	*P*-value
Age (year) Mean ± SD	11.49 ± 1.71	11.77 ± 2.30	0.330
Sex			
Male	41 (41.0%)	45 (45.0%)	0.568
Female	59 (59.0%)	55 (55.0%)	
Consanguinity	64 (64.0%)	30 (30.0%)	<0.001**
Height Z-score Mean ± SD	−1.36 ± 0.97	0.59 ± 0.75	<0.001**
Weight Z-score Mean ± SD	−0.97 ± 1.48	0.65 ± 0.56	<0.001**
BMI Z-score Mean ± SD	−0.74 ± 2.67	0.12 ± 0.76	0.002*
Hemoglobin (g/dl) Mean ± SD	7.19 ± 1.12	12.38 ± 1.39	<0.001**
Platelet count (× 10^9^/L) Mean ± SD	310.26 ± 129.37	235.74 ± 69.34	<0.001**
White blood cell count (× 10^9^/L) Mean ± SD	9.56 ± 5.03	8.39 ± 2.22	0.035*
HbA1c (%) Mean ± SD	6.12 ± 0.84	4.99 ± 0.66	<0.001**
Serum fructosamine (mmol/L) Mean ± SD	2.86 ± 0.45	2.34 ± 0.15	<0.001**
Fasting plasma glucose (mg/dl) Mean ± SD	103.37 ± 22.95	79.01 ± 7.38	<0.001**
Fasting insulin level (mIU/L) Mean ± SD	9.40 ± 2.96	5.04 ± 1.38	<0.001**
HOMA-IR Mean ± SD	2.32 ± 1.12	1.01 ± 0.36	<0.001**
HOMA-IS Mean ± SD	0.51 ± 0.23	1.20 ± 0.72	<0.001**

*HOMA-IR* Homeostatic Model Assessment for insulin resistance, *HOMA-IS* Homeostatic Model Assessment for insulin sensitivity, *SD* Standard deviation.

*Statistically significant at *p* < 0.05, **highly Statistically significant at *p* < 0.001.

According to FPG and 2-h post-prandial blood glucose (2-h PG), 49 patients (49%) were glucose tolerant, and 51 patients (51%) were glucose intolerant (32 patients had prediabetes state and 19 patients revealed diabetes mellitus). HbA1c, fructosamine, FPG level, FINS level and HOMA-IR were highly statistically increased in the glucose intolerant group compared to the glucose tolerant group (*p* < 0.001), furthermore HOMA-IS was statistically lower in glucose intolerant group compared to glucose tolerant group (*p* < 0.001). Also, serum ferritin, 6 months mean serum ferritin, blood transfusion index and poor compliance to chelation therapy were highly statistically increased in the glucose intolerant group compared to the glucose tolerant group (*p* < 0.001) Table 2.

**Table 2:** Comparison of demographic and laboratory data between glucose tolerant and glucose intolerant patients according to fasting plasma glucose and 2 h postprandial glucose levels.

Data	Glucose tolerant patients (*n* = 49)	Glucose intolerant patients (*n* = 51)	*P*-value
Age (year) Mean ± SD	10.88 ± 1.27	12.08 ± 1.89	<0.001**
≤10	20 (40.8%)	11 (21.6%)	
>10	29 (59.2%)	40 (78.4)	
HbA1c (%) Mean ± SD	5.58 ± 0.68	6.63 ± 0.62	<0.001**
Normal	27 (55.1%)	1 (2.0%)	
Pre diabetic	22 (44.9%)	29 (56.9%)	
Diabetic	0 (0.0%)	21 (41.2%)	
Serum fructosamine (mmol/L) Mean ± SD	2.56 ± 0.20	3.15 ± 0.44	<0.001**
Normal	49 (49.0%)	11 (11.0%)	
Abnormally high	0 (0.0%)	40 (40.0%)	
Fasting insulin level (mIU/L) Mean ± SD	8.14 ± 2.04	10.62 ± 3.21	<0.001**
HOMA-IR Mean ± SD	1.68 ± 0.56	2.94 ± 1.18	<0.001**
HOMA-IS Mean ± SD	0.65 ± 0.23	0.39 ± 0.15	<0.001**
Serum ferritin (μg/L) Mean ± SD	1730.22 ± 878.17	3209.82 ± 769.63	<0.001**
Mean serum ferritin among last 6 months (μg/L) Mean ± SD	1958.37 ± 963.16	3129.41 ± 785.89	<0.001**
Chelation duration (years) Mean ± SD	6.65 ± 1.44	7.33 ± 1.40	0.018*
Poor compliance to chelation	22 (44.9%)	9 (17.6%)	<0.001**
	27 (55.1%)	42 (82.4%)	
Transfusion index (ml/kg/year) Mean ± SD	247.06 ± 28.43	282.84 ± 48.44	<0.001**

*HOMA-IR* Homeostatic Model Assessment for insulin resistance, *HOMA-IS* Homeostatic Model Assessment for insulin sensitivity, *SD* Standard deviation.

*Statistically significant at *p* < 0.05, **highly Statistically significant at *p* < 0.001.

According to the normal reference range for fructosamine and HbA1c, glucose intolerance was documented in 40 patients (40%) with fructosamine compared to 72 patients (72%) with HbA1c. Of those glucose intolerant patients, 18 patients (18%) were diagnosed with diabetes mellitus based on FPG level and 2-h PG representing 45% by fructosamine detection compared to 25% by HbA1c detection. Only one patient in the normally glucose tolerant group had diabetes mellitus representing 1.67% by fructosamine detection compared to 3.57% HbA1c detection Table 3.

**Table 3:** Distribution of patients according to HbA1c and fructosamine results in relation to fasting blood glucose and 2-h post prandial blood glucose levels.

Data	Glucose tolerant patients	Prediabetes patients^a^	Patients with diabetes^b^	Total
	(*n* = 49)	(*n* = 32)	(*n* = 19)	(*n* = 100)
HbA1c (%)				
Normal (≤5.6%)	27 (55.1%)	0 (0.0%)	1 (5.3%)	28 (28%)
Pre diabetes (5.7–6.4%)	22 (44.9%)	25 (78.1%)	4 (21.1%)	51 (51%)
Diabetes (≥6.5%)	0 (0.0%)	7 (21.9%)	14 (73.7%)	21 (21%)
Serum fructosamine (mmol/L)				
Normal (2–2.85 mmol/L)	49 (100.0%)	10 (31.2%)	1 (5.3%)	60 (60%)
Abnormally high (>2.85 mmol/L)	0 (0.0%)	22 (68.8%)	18 (94.7%)	40 (40%)

^a^Prediabetic patients had fasting plasma glucose 100 mg/dl to 125 mg/dl or suffered impaired glucose tolerance (2-h postprandial blood glucose 140 mg/dl to 199 mg/dl).

^b^Diabetic patients had fasting plasma glucose ≥126 mg/dl or 2-h postprandial blood glucose ≥200 mg/dl.

**Fig. 1:**
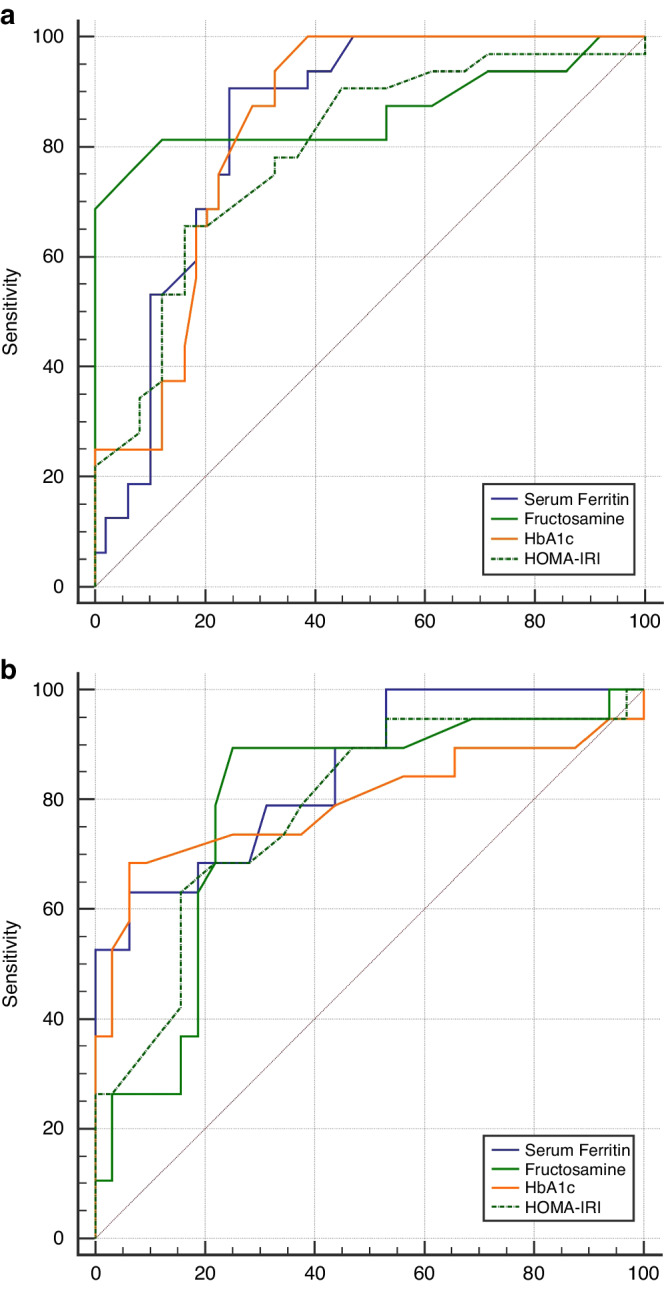
Receiver operating characteristic (ROC) curve for serum ferritin, fructosamine, hemoglobin A1c and insulin resistance index (HOMA-IR) in patients. (**a**) To discriminate between glucose tolerant and glucose intolerant patients (**b**) To discriminate between prediabetic and diabetic patients.

Furthermore, receiver operating characteristic (ROC) curve analysis demonstrated that a prediabetes state in patients was detected at a cut-off point >2.76 mmol/L for fructosamine with 81.3% sensitivity, 87.8% specificity, 81.2% positive predictive value (PPV) and 87.8% negative predictive value (NPV), and at a cut-off point >5.9% for HbA1c with 100.0% sensitivity, 60.4% specificity, 62.7% PPV and 100.0% NPV. On the other hand, diabetes mellitus was detected in patients at a cut-off point >3 mmol/L for fructosamine with 89.5% sensitivity, 75.0% specificity, 68.0% PPV and 92.3% NPV, and at a cut-off point >6.87% for HbA1c with 68.4% sensitivity, 93.8% specificity, 86.7% PPV and 83.3% NPV, Fig. 1.

In addition, HOMA-IR at a cut-off point >2.1 was found to detect a prediabetes state in patients with 65.6% sensitivity, 83.7% specificity, 72.4% PPV and 78.8% NPV, and at a cut-off point >3.2 was found to detect diabetes mellitus affection with 63.2% sensitivity, 84.4% specificity, 70.6% PPV and 79.4% NPV. Also, HOMA-IR showed a significant positive correlation to age, HbA1c, fructosamine, FPG level, FINS level, and serum ferritin and showed a significant negative correlation to HOMA-IS. On the other hand, there was a strong significant negative correlation between HOMA-IS and age, HbA1c, fructosamine, FPG level, FINS level, HOMA-IR and serum ferritin Table 4 and Fig. 1.

**Table 4:** Correlation between serum ferritin level and other studied parameters in patients.

Data	Serum ferritin (μg/L)		HOMA-IR		HOMA-IS	
	*r*	*P*-value	*r*	*P*-value	*r*	*P*-value
Age (year)	0.424	<0.001**	0.442	<0.001**	−0.375	<0.001**
BMI (kg/m2)	0.259	<0.001**	0.218	0.03*	−0.243	0.015*
HbA1c (%)	0.598	<0.001**	0.390	<0.001**	−0.405	<0.001**
Serum fructosamine (mmol/L)	0.580	<0.001**	0.506	<0.001**	−0.531	<0.001**
Fasting insulin level (mIU/L)	0.315	0.001*	0.739	<0.001**	−0.660	<0.001**
HOMA-IR	0.563	<0.001**			−0.886	<0.001**
HOMA-IS	−0.474	<0.001**	−0.886	<0.001**		
Fasting plasma glucose (mg/dl)	0.609	<0.001**	0.653	<0.001**	−0.626	<0.001**
2 h post prandial glucose (mg/dL)	0.393	<0.001**	0.328	0.001*	−0.309	0.002*
Serum ferritin (μg/L)			0.480	<0.001**	−0.489	<0.001**
Chelation duration (years)	0.175	0.082*	0.304	0.002*	−0.217	0.030*
Transfusion index (ml/kg/year)	0.234	0.019*	0.133	0.187	−0.141	0.161

*HOMA-IR* Homeostatic Model Assessment for insulin resistance, *HOMA-IS* Homeostatic Model Assessment for insulin sensitivity.

*Statistically significant at *p* < 0.05, **highly Statistically significant at *p* < 0.001.

**Fig. 2:**
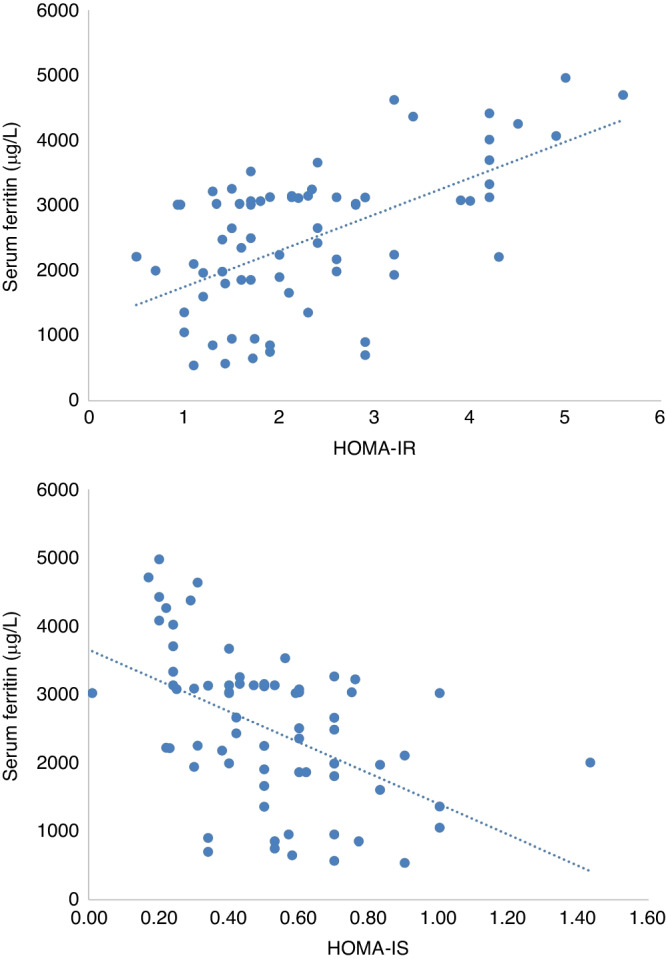
Correlation between serum ferritin and insulin resistance index (HOMA-IR), and insulin sensitivity index (HOMA-IS).

A significant positive correlation was found between serum ferritin and age, body mass index, HbA1c, fructosamine, FPG level, 2-h PG, HOMA-IR and blood transfusion index. There was a significant negative correlation between serum ferritin and HOMA-IS. Furthermore, serum ferritin at a cut-off point >2101 μg/L was associated with a prediabetes state with 90.9% sensitivity, 75.5% specificity, 70.7% PPV and 92.5% NPV, and at a cut-off point >3325 μg/L was associated with diabetes mellitus with 63.2% sensitivity, 93.8% specificity, 85.7% PPV and 81.1% NPV, Table 4 and Figs. 1, 2.

## Discussion

Beta thalassemia major is associated with chronic iron overload due to extravascular hemolysis, increased iron absorption, dysfunctional iron usage, and long-term transfusion therapy, resulting in serious consequences.^12^ Blood glucose dysregulation has been related to the effect of iron overload on the different tissues controlling the carbohydrate homeostatic mechanisms, including the pancreas and liver.^13^

The use of HbA1c to identify the problems in blood glucose levels in β-TM patients has been debatable. The role of fructosamine over HbA1c has been highlighted in β-TM patients being analyzed in a similar way to serum proteins rather than HbA1c which might get affected in thalassemia by other factors, such as erythropoiesis, hemoglobin disease, glycosylation levels, and erythrocyte longevity.^14^

According to the American Diabetes Association, diagnosis of diabetes mellitus could be made with HbA1c ≥ 6.5% and prediabetes range at HbA1c 5.7% to 6.4%. Furthermore, the normal ranges for fructosamine in non-diabetes mellitus individuals are 2.0 to 2.85 mmol/L.^14^ Unlike HbA1c, there is no reference point for fructosamine at which diabetes mellitus diagnosis could be made.

In this study, HbA1c and fructosamine were statistically significantly higher in β-TM patients compared to controls and in the glucose intolerant patients than in glucose tolerant ones. Glucose intolerance was documented in 72% of patients with HbA1c % ≥5.7, compared to 40% with fructosamine >2.85 mmol/L, in both 18 patients (18%) were confirmed to have diabetes mellitus based on FPG level and 2-h PG, representing 25% with HbA1c detection compared to 45% with fructosamine detection.

Our results documented HbA1c cut-off point >5.9% to detect prediabetes state and >6.87% to detect established diabetes mellitus in our patients which were close to known reference ranges. On the other hand, fructosamine levels documented a cut-off point >2.78 mmol/L to detect prediabetes state and a cut-off point >3 mmol/L to detect established diabetes mellitus in our patients recognizing that no established fructosamine reference range indicates whether a person has diabetes or not. Furthermore, fructosamine was more specific with a higher PPV in detecting prediabetes state and more sensitive with a higher NPV in identifying diabetes mellitus in our patients when compared to HbA1c.

A comparable study in adults by Malmström et al.^15^ determined that a threshold level of 2.5 mmol/L of fructosamine was found to be effective in differentiating subjects with and without diabetes. Selvin et al.^16^ reported an adjusted hazard ratio of 4.96 for incident diabetes for those with fructosamine levels >2.64 mmol/L compared to those below 2.41 mmol/L.

An adult study by Choudhary et al.^17^ and a pediatric study by Gomber et al.^13^ pointed out that β-TM patients may have higher baseline values of HbA1c limiting its diagnostic ability for diabetes mellitus. Despite this, HbA1c continues to be a good marker for worsening glucose homeostasis. A pediatric study by Ji et al.^18^ and an adult study by Gluvic et al.^19^ suggested measuring HbA1c using a different technique, such as the Capillary’s 2 Flex Piercing System, or measuring HbA1c and fructosamine at the same time to evaluate glycemic status in β-TM patients. A pediatric study by Kosaryan et al.^20^ reported that mean fructosamine had a substantial correlation with blood glucose and was a more reliable measure of retrograde glycemic levels than HbA1c in individuals with β-TM.

Other studies in adults and children illustrated that for evaluating glycemic control over an extended period (two to three months), serum HbA1c is the preferred metric, however, fructosamine may be a valuable and reliable diagnostic biomarker and of exceptional use in some conditions when HbA1c is insufficient. Additionally, as it offers data on short-term glycemic control, it is anticipated to represent poor glycemic control more accurately than HbA1c.^21,22^

In this study, insulin resistance index (HOMA-IR) and fasting insulin levels were statistically significantly higher in patients than in controls and glucose intolerant patients when compared to normal ones. Furthermore, insulin sensitivity index (HOMA-IS) was statistically significantly less in patients than in controls and in glucose intolerant patients than in normal ones. Therefore, insulin resistance in β-TM patients is the cornerstone for abnormal glucose tolerance than insulin deficiency.

Similarly, various studies in pediatrics reported that HOMA-IR was greater in β-TM patients who either had diabetes mellitus or prediabetes in comparison to the euglycemic patients. Others highlighted that acute increases in serum ferritin following blood transfusion in those patients may contribute to an increase in insulin secretion and insulin resistance.^6,23^ Gomber et al.^13^ observed that β-TM children with HOMA-IR ≥ 2.5 were risk 16.4 times of developing impaired glucose tolerance. Therefore, various studies in children highlighted the importance of frequently assessing blood glycemic status in β-TM patients, especially those above the age of 10 who should receive an annual FPG level and oral glucose tolerance test (OGTT), for early diagnosis and active intervention of glucose intolerance.^2,6,24^

Iron overload adversely affects glucose metabolism. It has been observed that the incidence of diabetes mellitus in children with serum ferritin >2500 μg/L was 3.53 times higher than that in patients with serum ferritin between 1000–2500 μg/L, and the reduction of serum ferritin could improve the secretion function of beta cells.^25^

In this study, serum ferritin was statistically significantly higher in glucose intolerant patients compared to normal ones, and in patients with diabetes compared to prediabetes or glucose tolerant patients. There was a statistically significant positive correlation between serum ferritin and age of patients, HbA1c, fructosamine, FPG level, 2-h PG, FINS level, HOMA-IR, and transfusion index. Furthermore, our results determined that at a cut-off point >3325 μg/L for serum ferritin, β-TM patients are more liable to have diabetes mellitus.

In the same context, Liang et al.^26^ demonstrated that β-TM children with impaired fasting glucose levels were significantly older and had higher FINS, HOMA-IR, and a significantly lesser HOMA-IS than those with normal fasting glucose levels.

The limitation of this study was a small sample size so; further large-scale prospective studies need to be carried out in this regard to validate the results of this study.

## Conclusion

Overt and preclinical abnormal glucose tolerance is relatively common in β-TM, mainly due to developing insulin resistance with their prevalence increasing with age or longer duration of the disease and poor compliance to chelation therapy. HbA1c and fructosamine are valuable in evaluating the blood glucose status in β-TM children, and however, HbA1c is a reliable indicator of deteriorating glucose metabolism, fructosamine is an uprising alternative indicator of glucose status, which requires further studies for confirmation. Finally, patients should periodically check their blood glucose status and their chelation therapy should be improved with good compliance.

## Data Availability

All datasets presented in this study are included in the article.
